# Distinct behavioral and brain changes after different durations of the modified multiple platform method on rats: An animal model of central fatigue

**DOI:** 10.1371/journal.pone.0176850

**Published:** 2017-05-11

**Authors:** Chenxia Han, Feng Li, Jie Ma, Yan Liu, Weihong Li, Yingqiu Mao, Yuehan Song, Siyuan Guo, Jing Liu

**Affiliations:** 1 Basic Medicine School, Beijing University of Chinese Medicine, Beijing, People’s Republic of China; 2 Science Research Center of Chinese Medicine, Beijing University of Chinese Medicine, Beijing, People’s Republic of China; Universidade de Sao Paulo, BRAZIL

## Abstract

The modified multiple platform method (MMPM) is a classical sleep deprivation model. It has been widely used in behavioral and brain research, due to its effects on physical and mental functions. However, different MMPM protocols can promote distinct effects in rats. Although the MMPM has been proved to induce central fatigue, the effects of different durations of subjection to the MMPM remain undetermined. This study aims to investigate the changes in behavior, N-Methyl-d-Aspartate receptor 1 (NR1) and 2A (NR2A), as well as the ultrastructural alteration in the hippocampus after different MMPM modelling, to compare the central fatigue effect induced by dynamic MMPM. Rats were randomly divided into four groups: 5-, 14- and 21- day MMPM groups, and a control group. Each MMPM group underwent a 14-hour daily MMPM modelling. After each training session, open field and elevated plus maze tests were performed. Corticosterone levels were detected by ELISA, and the hippocampal NR1 and NR2A were measured by RT-PCR and Western blot analysis. In addition, ultrastructural changes in the hippocampal cornu ammonis 1(CA1) region were determined by transmission electron microscopy (TEM). The findings showed that the 5 and 14 days of MMPM induced a high-stress state, while the 21 days of MMPM induced anxiety and degenerative alteration in the hippocampal morphology. Additionally, hippocampal NR1 and NR2A gene expression decreased in all MMPM groups, whereas the protein expression only decreased in the 21-day group. Overall, different durations of MMPM caused distinct behavioral and brain changes, and the 21 days of MMPM could induce central fatigue.

## 1. Introduction

A close relationship between the rhythm and duration of sleep and the central nervous system (CNS) function has been found[1]. Mental and physical functions can be affected by sleep deprivation. In humans, chronic sleep deprivation can result in anxiety[2], and decreased ability of learning and memory[3–4]. Furthermore, continuous sleep deprivation could induce fatigue and even severe disease[5]. The modified multiple platform method (MMPM) has been extensively applied in sleep deprivation of rodents[6]. The equipment consists of a plastic tank and several platforms, which are stuck to the bottom of the tank. When the tank is filled with water (1-2cm to the surface of platform), rats are forced to stand on the platforms because of their instinctive fear of water, when they fall asleep, they will fall into the water and wake up[7]. This method can effectively affect emotion[8–9] and cause cognitive dysfunction[10]. Central fatigue is defined as the failure to initiate and/or sustain attentional tasks and physical activities requiring self-motivation (as opposed to external stimulation)[11–12]. As is different from peripheral fatigue, central fatigue is a condition involving a decrease in brain high level cognition and negative emotions such as anxiety[13]. Previous studies found that the intermittent subjection to MMPM could induce central fatigue[14–15], and the condition could be treated by CNS function-improving medicine [16–17]. However, research on this topic remains scant and needs to be enriched.

There are three criteria in animal models for human disorders of rodents. Construct validity (i.e., derived from similarity in the underlying mechanisms—physiological or psychological), face validity (i.e., phenomenological similarity between human clinical condition symptoms and symptoms expressed in the animal model), and predictive validity (i.e., an animal’s response to medication can predict human response) [18–19]. Sleep deprivation could directly decrease CNS function, and the condition produced by MMPM generally meets the manifestation of central fatigue (cognitive dysfunction and negative emotion). In addition, treatments that enhance CNS function can effectively improve the MMPM condition. These evidence shows that the MMPM may meet the validation criteria of the animal model of central fatigue.

Although numerous studies have investigated the effect of MMPM on CNS function and behavioral parameters, such as learning ability[20], anxiety[21], and manic behavior[22], most of them studied the continuous MMPM modelling from 24h to 96h. Only a few studies have investigated intermittent MMPM modelling, such as for 5 and 21 days, without systematic contrast or further classification. Moreover, the effect of sleep deprivation on CNS function is not unidirectional; sleep deprivation may induce a positive or negative effect depending on certain factors, such as duration[23]. As a result, the central fatigue model induced by MMPM has become difficult to construct and evaluate.

Glutamate (Glu) is a classical excitatory neurotransmitter. It is closely related to cognition, emotion, stress, and fatigue[24]. NR1 and NR2A are two N-methyl-D-aspartate (NMDA) receptor subtypes of Glu, they both participate in the process of synaptic plasticity, and various psychiatric disorders, such as anxiety and depression[25]. The hippocampus is the dominant brain region that processes cognition and emotion. In addition, NR1 and NR2A can regulate the synaptic plasticity of hippocampal neurons[26].Thus, the current work aims to analyze the changes in behavior, NR1 and NR2A, synapses and mitochondria, after a gradient increase in the duration of intermittent MMPM on rats, in order to construct an effective model of central fatigue.

## 2. 2. Material and methods

### 2.1 Animal

Adult male Wistar rats (weighing 160–180g) were purchased from Beijing Vital River Laboratory Animal Technology Limited Company (Beijing, China). Animals were kept in a room at a constant temperature of 23 ± 1°C, relative humidity of 30% to 40%, light-dark cycle of 12 h (from 06:00 to 18:00), and ad libitum supply of food and purified water. Rats were individually adapted in home cages and adapted to the new environment in the experiment room for 7 days. Thirty-two rats were randomly divided into four groups as follows: 5-day group (n = 8), 14-day group (n = 8), 21-day group (n = 8) and control group (n = 8).

The experiments were approved by the Institutional Animal Ethics Committee of Beijing University of Chinese Medicine. All the animals were maintained in accordance with the guidelines outlined by the Chinese legislation on the ethical use and care of laboratory animals. Moreover, all efforts were made to minimize animal suffering and the number of animals used to produce reliable data.

### 2.2 MMPM

Fifteen plastic platforms were fixed at the bottom of a plastic tank (110 × 60 × 40cm). The tank was filled with water at a temperature of 20°C to 25°C and a depth of 1.0cm below the platform surface. Iron cages and bottles were filled with food and water, respectively, hanging on the tank cover (iron gauze wire netting). The 5-, 14- and 21-day groups were subjected to MMPM. All MMPM protocols started on the following day and lasted from 18:00 to 8:00. Each rat was subjected to MMPM for 14h per day. Rats in the 5-day group were subjected to MMPM for 5 consecutive days, and rats in the 14- and 21-day groups were subjected to MMPM for 14 and 21 consecutive days, respectively. The rats were returned to their home cages after being subjected to the 14-h MMPM, at 8:00am and supplied with food and purified water. The tank for the MMPM was cleaned carefully by experimenters, and filled with water at a temperature of 20°C to 25°C before 18:00 pm. Rats were carefully monitored to avoid accidental injury during training and placed in individual cages after the MMPM. The MMPM was performed in a separate room which had the same environment.

The rats in the control group were routinely fed for 21 days.

### 2.3 Behavioral tests

The behavioral tests were performed on the following day after each training section (for instance, the behavioral tests of 5-day group were performed directly after 5 days of MMPM training). The tests were initiated at 8:00 am in the following sequence: open field test (OFT) and elevated plus maze (EPM). Each rat was transported to the test room 2 h prior for acclimatization, a 1.5-hour break was given for all rats between the two tests. Behavioral tests were analyzed using the EthoVision XT software (Noldus, the Netherlands). Each rat was placed in the central square in both tests and was tested once only in each test. The open field arena and the arms of the EPM were thoroughly cleaned with 75% ethanol between rats during each test.

#### 2.3.1 Open field test(OFT)

The OFT provides a novel environment in which to measure animal locomotion and emotion[27]. The open field arena (100 ×100 × 40 cm) is made of acrylic, with grey walls and a black floor, and is divided into 25 equally sized areas. Each rat was tested for 3 min. Five parameters were tested: 1) the time spent in central (60 × 60 cm); 2) the total distance travelled; 3) the maximum distance travelled during an uninterrupted run; 4) the mean velocity; 5) the tracking during the test period (recorded by a camera and drawn using a computer software).

#### 2.3.2Elevated plus maze(EPM)

The EPM is a classical paradigm for evaluating anxiety in rodents[28].The EPM in the current study was constructed as in previous studies: with two open arms (30 × 5 × 15 cm) and two closed arms (30 × 5 × 15 cm) that extended from a central, open square (5 × 5 cm) [29–30]. The maze was elevated on a pedestal to a height of 45 cm above the floor. Each rat was tested for 3 min. Two parameters were tested: 1) the amount of time to explore the open arms relative to the total amount of time to explore the open and closed arms of the maze, recorded as the ratio of time spent in the open arms/time in the arms; 2) the total number of entries into the open arms relative to the total entries, recorded as the ratio of number of open arms entries/total entries.

### 2.4 Transmission electron microscope(TEM)

Rats were deeply anaesthetized with an intraperitoneal injection of 10% pentobarbital sodium (0.40 mL/100 g body weight), then immediately sacrificed by rapid decapitation. All rats were comfortably sacrificed under anesthesia. After removing the hippocampus, three randomly chosen pieces of the CA1 region of the hippocampus were cut into 1mm^3^ and immediately fixed in 2.5% glutaraldehyde (pH = 7.4) at 4°C for 4h. The samples were then dehydrated and fixed according to previous studies[31]. Fixed samples were observed and photographed by TEM (JEM1230, JATAN, Japan).

### 2.5 Enzyme-linked immunosorbent assay (ELISA)

Trunk blood was collected in a blood collection tube and centrifuged at 3,000 rpm at 4°C for 20min. The obtained serum was then used to detect corticosterone. An ELISA kit was used to measure corticosterone level (Enzo Life Science, Inc., Lausen, Switzerland).

### 2.6 Real-time PCR(RT-PCR) analysis

The hippocampus was removed and immediately placed in liquid nitrogen and then stored at −80°C until assayed. Gene expression of NR1 and NR2A was analyzed by quantitative RT-PCR. Total RNA was extracted from the hippocampus using the SV Total RNA Isolation System (Promega Z3100, Fitchburg, USA). The total RNA (1μg) was reverse-transcribed to 50μl cDNA using a Reverse Transcription System (Promega A3500). Both methods were performed according to the manufacturer’s instructions. Primers were designed using Primer-BLAST (NCBI, Bethesda, MD, USA) according to the mRNA sequences (in GenBank) for NR1 (NM_ 017010), NR2A (NM_ 012573) and β-actin (NM_031144.2, as control). The PCR products were run on a 0.8% agarose gel to confirm that the products were of the expected size. Results were normalized against β-actin expression. The primer sequences are listed in Table 1. The Real-time quantitative PCR analysis were performed using the CFX96 Touch instrument (Bio-Rad, Hercules, CA, USA).

**Table 1:** The primers for RT-PCR.

Genes	Primers
NR1	5’-GGACTGACTACCCGAATGTCCA-3’(forward)
	5’-GTAGACTCGCATCATCTCAAACCA-3’(reverse)
NR2A	5’-GCTTGTGGTGATCGTGCTGAA-3’(forward)
	5’-AATGCTGAGGTGGTTGTCATCTG-3’(reverse)
β-Actin	5’-GGAGATTACTGCCCTGGCTCCTA-3’(forward)
	5’-GACTCATCGTACTCCTGCTTGCTG-3’(reverse)

The reaction conditions were as follows: initial activation at 95°C for 30 s; 40 cycles of denaturation at 95°C for 5 s, annealing at 60°C for 30s; and melt curve determination at 65°C to 95°C for 5 s. All the measurements were performed in triplicate. The RT-PCR data are presented as Ct values. The relative expression levels of NR1 and NR2A were determined using the 2-ΔΔCt method[32].

### 2.7 Western blot analysis

Hippocampus was lysed using a Mem-PER Eukaryotic Membrane Protein Extraction Reagent Kit (Pierce Biotechnology, Rockford, IL, USA). Protein concentration was determined by the BCA assay kit (Solarbio Life Science, Beijing, China). Equal amounts of lysates were separated on a SDS-12% PAGE and then transferred onto polyvinylidene difluoride(PVDF) membranes (10μl each sample, 80 V, 2.5 h). After blocking with 5% dried skim milk for 2 h, the membranes were incubated with Anti-NMDAR1 antibody (Rabbit polyclonal to NMDAR1, Abcam, Cambridge, UK. 1:1000), Anti-NMDAR2A antibody (Rabbit polyclonal to NMDAR2A, Abcam, 1:1000) and Anti-actin antibody (Rabbit polyclonal to beta Actin, Abcam, 1:6000) antibodies at 4°C overnight. The samples were then incubated with IRDyeTM secondary antibodies (1:10,000) for 1 h in room temperature. Protein bands were visualized on a LiCor Odyssey instrument (LiCor Biotechnology, Lincoln, NE, USA).

### 2.8 Statistic analysis

Data were analyzed with the Statistical Package for the Social Sciences (SPSS) version 17.0 (IBM, Corp., NY, USA) and expressed as the mean ± standard error of mean (SEM). All the data were initially tested for normality and homogeneity of variance, and then, one-way ANOVA or Kruskal–Wallis was applied. In addition, the least significant difference method or Mann–Whitney test was adopted for comparison between groups. P values < 0.05 were considered statistically significant.

## 3. Results

### 3.1 OFT

**Fig 1:**
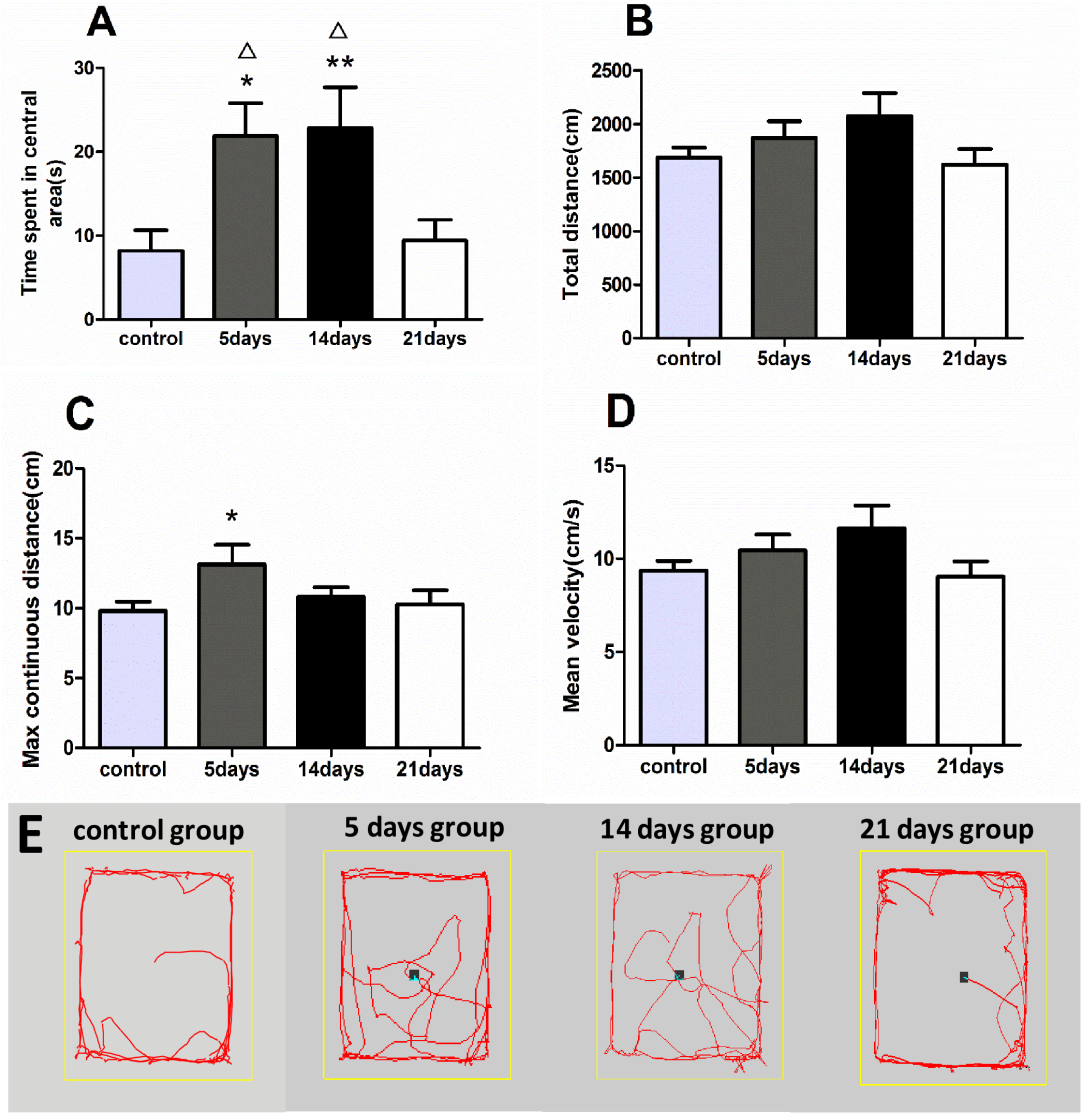
Analysis of OFT. All data are presented as the mean ± SEM (n = 8); * refers to p <0.05 vs. control group. Δ refers to p<0.05 vs. 21-day group **A:** The time spent in the central area was recorded by a test lasting 3min. The time spent in the center of the 5- and 14-day group increased compared with that of the control group. **B:** No significant difference in the total distance travelled was observed among the four groups. **C:** A significant difference in the maximum continuous distance travelled was found among the four groups, and the 5-day group travelled a longer continuous distance compared with the control group. **D:** The mean velocity showed no significant difference among the four groups. **E:** Representative track of every group.

The time spent in the center relates to the anxiolytic-like behavior of rodents[33]. Based on one-way ANOVA tests, the time spent in the center was significantly different among the four groups [F(3,28) = 4.795, p<0.01]. This parameter of the 5- and 14-day groups both increased compared with the control group (p<0.05). The total distance travelled, maximum distance travelled during an uninterrupted run (maximum continuous distance) and mean velocity reflect the locomotion behavior of rats. One-way ANOVA test showed no significant difference in the total distance travelled [F(3,28) = 1.651, p>0.05]. However, the Kruskal–Wallis test revealed a significant difference in the maximum continuous distance travelled among the four groups [H(3) = 9.397, p<0.05]. The 5-day group travelled a longer continuous distance compared with the control group (p<0.05). The mean velocity, another indicator of locomotion activity, showed no significant difference among the groups [F(3,28) = 1.716, p>0.05]. The representative track of every group revealed that the 5- and 14-day MMPM evidently increased the central travelling compared with control group. (Fig 1, data in S1 File. Raw data of OFT).

### 3.2 EPM

**Fig 2:**
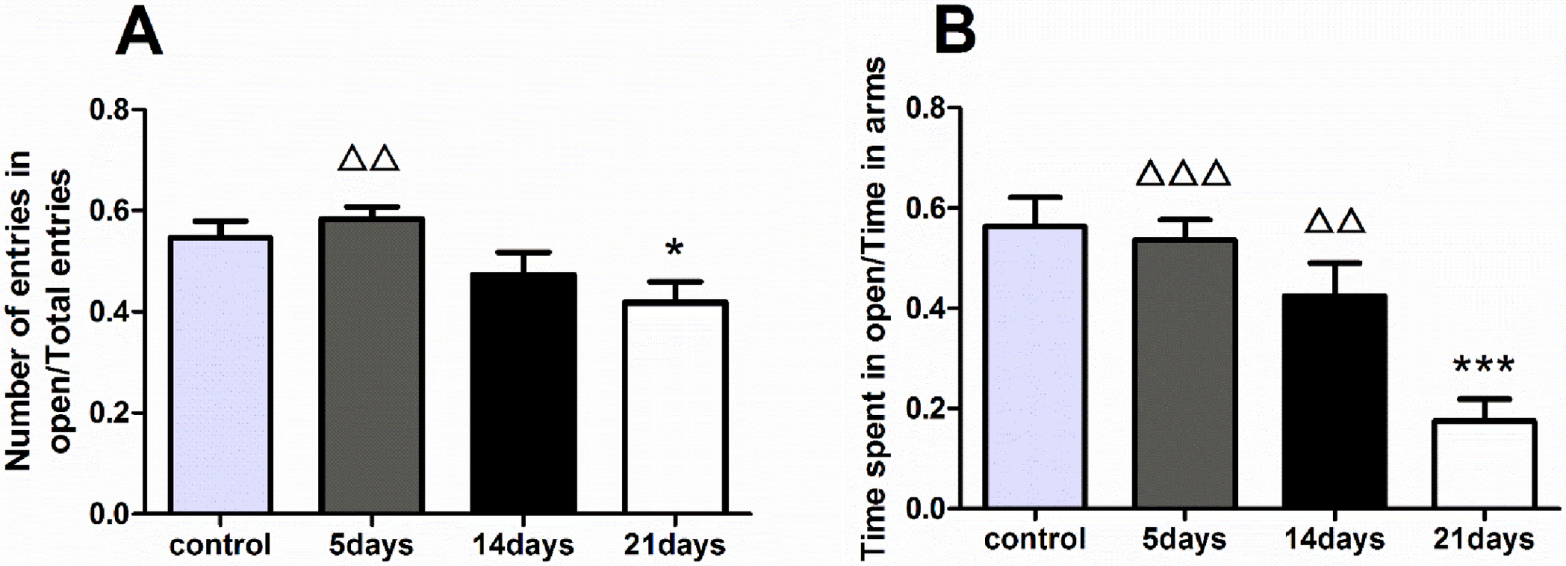
Analysis of EPM. All data are presented as the mean ± SEM (n = 8); * refers to p <0.05 vs. control group. ΔΔ refers to p<0.01, ΔΔΔ refers to p<0.001 vs. 21-day group. **A:** The ratio of the number of entries in the open arms/total entries showed a significant difference among the four groups, and the index decreased significantly in the 21-day group compared with the control group. **B:** The ratio of the time spent in the open arms/all arms showed a significant difference among the four groups, and consist with the previous result, the 21-day MMPM significantly decreased the time spent in the open arms compared with the control group.

Two parameters measured by this test are used to evaluate anxiety in rats. One is the amount of time in the open arms relative to the total amount of time that the rat explored both in the open and closed arms, expressed as a ratio. One-way ANOVA test revealed a significant difference among the four groups in this parameter [F(3,28) = 11.023, p<0.001], and the 21-day group spent less time compared with the control group (p<0.001). The other parameter is the number of open arm entries relative to the total number of entries into both arms, expressed as a ratio. A significant difference was observed among the four groups based on the one-way ANOVA test [F(3,28) = 3.953, p<0.05]. As with the previous result on the first measure, the rats of the 21-day group visited the open arm less frequently compared with those of the control group (p<0.001). (Fig 2, data in S2 File Raw data of EPM).

### 3.3 Corticosterone level

**Fig 3:**
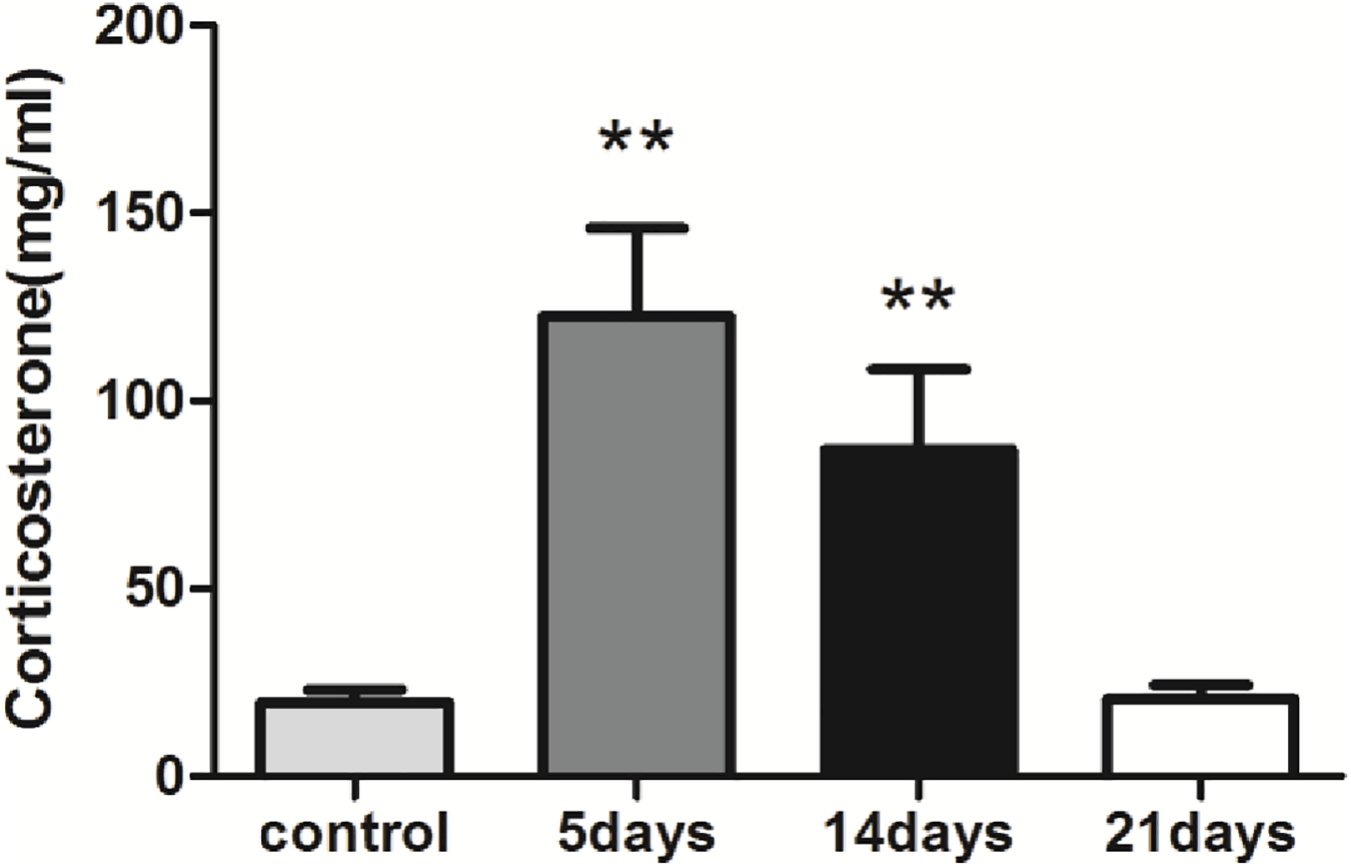
Increasing corticosterone level in blood serum of 5- and 14- groups. All data are represented as the mean ± SEM (n = 8); ** refers to p<0.01 vs. control group. A significant difference in corticosterone level was observed among the four groups. Compared with the control group, the 5- and 14-day groups have significantly increased corticosterone levels.

The analysis of blood serum corticosterone concentration revealed a significant difference among the four groups based on the Kruskal–Wallis test [H(3) = 18.855, p<0.001]. Both the 5- and 14-day groups showed higher corticosterone concentrations compared with the control group (p<0.01). However, no significant difference was found between the 21-day and the control group (p>0.05) (Fig 3, data in S3 File Raw data of Corticosterone).

### 3.4 Expression of mRNA of NMDA receptor (NR1 and NR2A)

**Fig 4:**
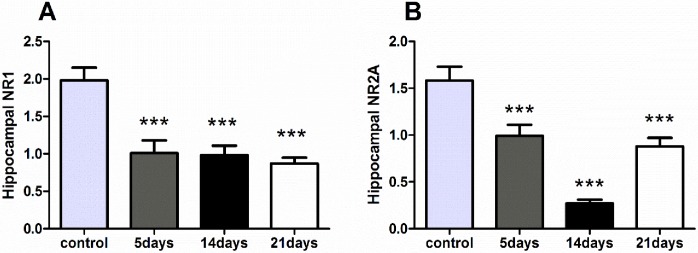
Decreasing NR1 and NR2A gene expression in 5-, 14- and 21-day groups. All data are presented as the mean ± SEM (n = 8); *** refers to p <0.001 vs. control group. The expression of NR1 **(A)** and NR2A **(B)** in the hippocampus of each MMPM group showed a significant decrease compared with the control group.

For the RT-PCR analysis of gene expression in the hippocampus, NR1 expression showed a significant difference among the four groups [F(3,28) = 13.074, p<0.001]. Each group exhibited a decrease in NR1 mRNA expression as compared with the control group (p<0.001). A significant difference was observed in hippocampal NR2A mRNA expression among the four groups [F(3,28) = 24.432, p<0.001]. Like the previous result on NR1, MMPM caused a decrease in NR2A expression at all durations, compared with the control group(p<0.001) (Fig 4, data in S4 File Raw data of NR1 and NR2A).

### 3.5 Protein expression of NMDA receptor (NR1 and NR2A)

**Fig 5:**
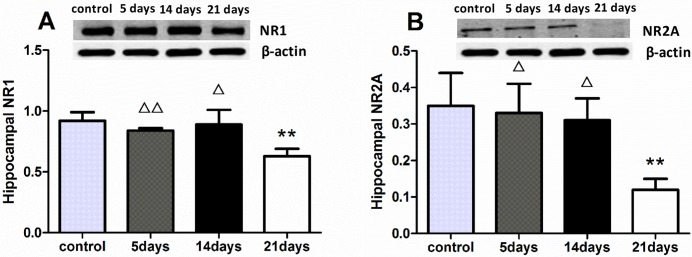
Decreased NR1 and NR2A protein expression in 21-day group. All data are presented as the mean ± SEM (n = 8); **refers to p<0.01 vs. control group. Δ refers to p<0.05, ΔΔ refers to p<0.01 vs. 21-day group. A representative blot and quantitation of the NR1/NR2A relative to β-actin ratio are shown. The molecular weight, based on the band size of NR1, NR2A, and β-actin were 120kDa, 170kDa, and 50kDa respectively. The levels of both the NR1 **(A)** and NR2A **(B)** proteins of the 21-day group decreased compared with the control group, and the other two groups as well.

The Western blot analysis revealed a significant difference in the level of NR1 protein among the groups [H(3) = 12.006, p<0.01]. Compared with the control group, the level of NR1 in the 21-day MMPM group was significantly decreased(p<0.01), while no change was detected in the 5- and 14-day groups(p>0.05). Similarly, a significant difference was detected in the NR2A [H(3) = 9.139, p<0.05]. The 21-day group exhibited a significant decrease compared with the other three groups (Fig 5, data in S4 File Raw data of NR1 and NR2A).

### 3.6 Ultrastructural changes in the CA1 region of the hippocampus

**Fig 6:**
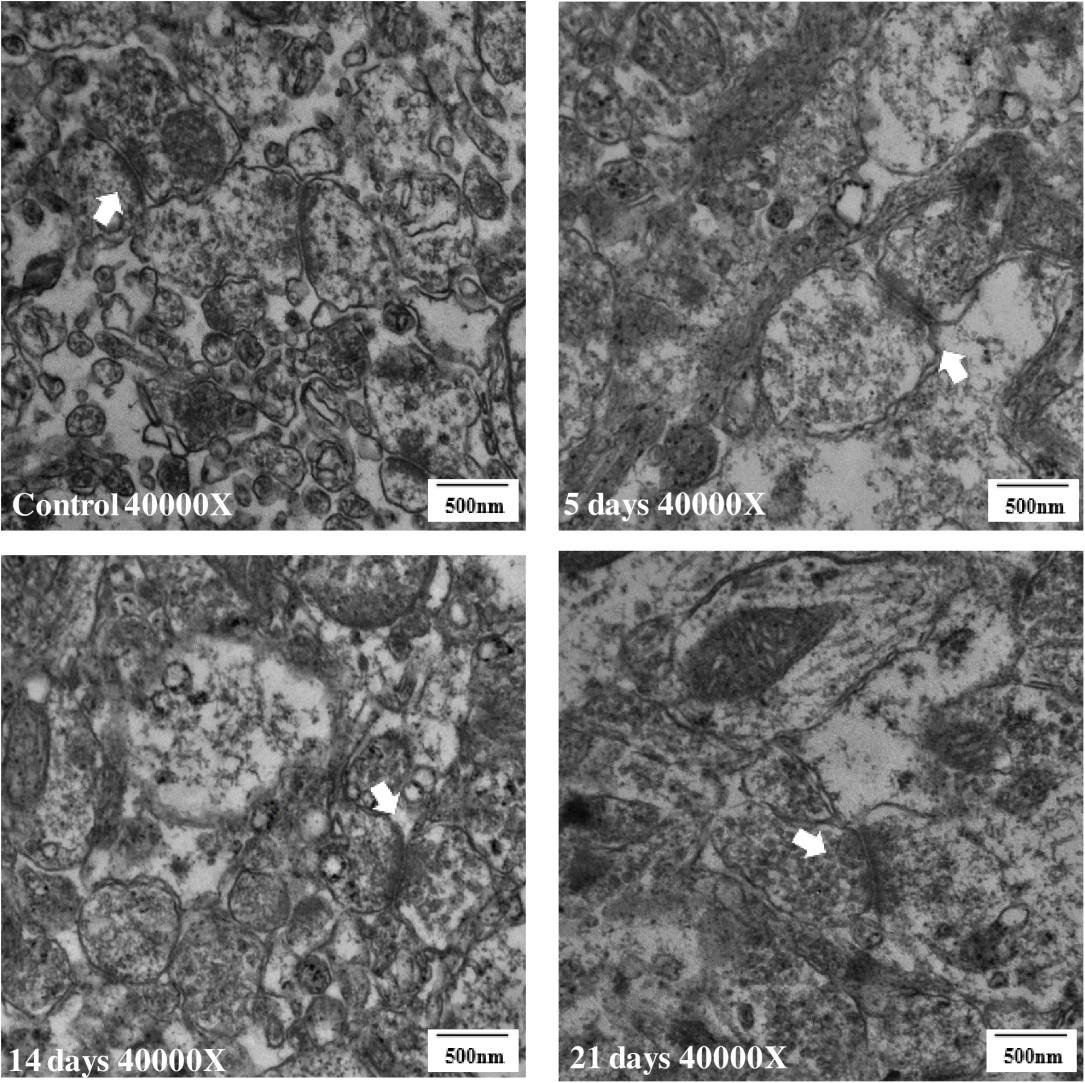
Degenerative changes of hippocampal synapses in 21-day group(40,000×) (n = 3). Compared with the control group (A), the synapses of the 5-day group (B) and 14-day group (C) did not show any evident change. The synaptic cleft of the 21-day group (D) was wider, and the synaptic vesicles were reduced.

The ultrastructural micrographs of the hippocampal synapses in the CA1 region of the four groups are shown in Fig 6 (pictures in S6 File TEM pictures). The hippocampal synapses in the 21-day group exhibited a degenerative change compared with the control group (Fig 6A). The synapses showed wider synaptic cleft between the two synaptic membranes and decreased synaptic vesicles (Fig 6D). However, no evidence of change in the synapses was observed in the 5 and 14-day groups (Fig 6B and 6C).

**Fig 7:**
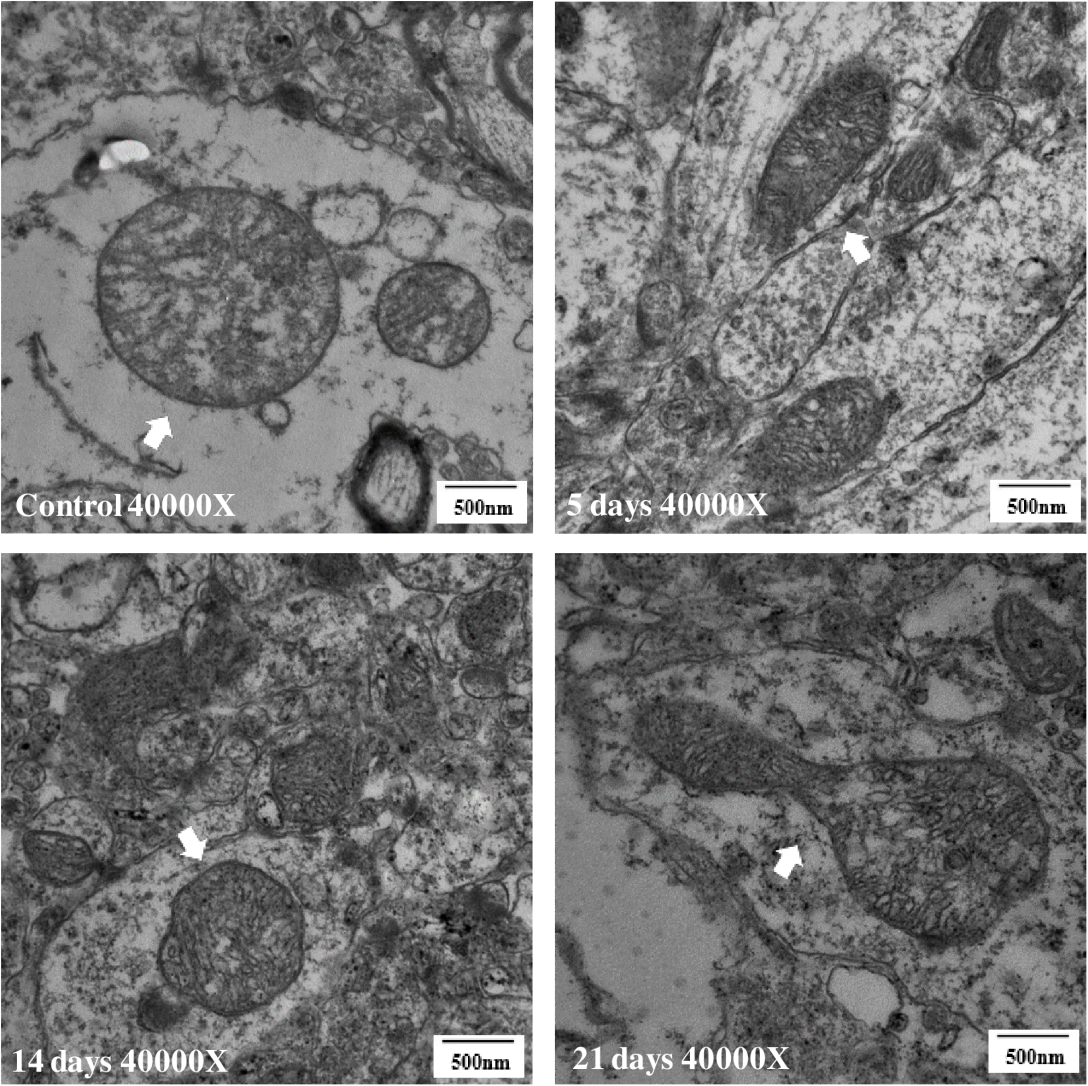
Degenerative changes of hippocampal mitochondria in 21-day group(40,000×) (n = 3). Compared with the control group (A), the 5-day group (B) and 14-day group (C) showed no visible changes, whereas the mitochondria of the hippocampus of the 21-day group (D) showed degenerative changes such as a stretched morphology and a ruptured double membrane.

Fig 7 shows the ultrastructural changes in the mitochondria of hippocampus. A rupture mitochondrial cristae and double membrane were observed in the 21-day group (Fig 7D), whereas no evidence of change was observed in the 5- and 14-day groups compared with the control group (Fig 7B and 7C).

## 4. Discussion

Sleep deprivation significantly influences the occurrence of central fatigue. Generally, rats need 12.6 h of sleep per day[34–35], approximately 2.4–4.2 h (20%–35% of total sleeping time) during night and 8.2–9.6 h (65–80% of total sleeping time) at daytime[36–37]. During the MMPM modelling, sleep was deprived because rats fell into the water and woke up when they fell asleep on the platform. Moreover, other factors of the MMPM, such as tedious and repeated environment and long-term standing on the platform, contributed to central fatigue. These factors caused behavioral and brain changes as well. Since central fatigue is a phenomenon induced by multiple factors, we attempt to put other factors into the modelling, such as physical output and emotional interference, to increase the construction validity. Additionally, based on our previous research, the duration of sleep deprivation in this study was set to 14h, from 18:00 to 8:00, whereby sleep deprivation and physical output can be both considered, instead of simple sleep deprivation. Although the MMPM has been used to generate central fatigue, intermittent duration and the time point have not been considered or introduced as factors. The present study proved that different MMPM protocols can generate distinct behavioral and brain changes, 21 days of MMPM could generate substantive anxiety without stress response, degenerative changes in hippocampal synapses and mitochondria, and down regulation of NMDA receptors, which make the condition favorable to develop central fatigue. This research is the first study to detect the dynamic change by intermittent MMPM. It could be used as supportive evidence for the development of central fatigue animal model, and the basis of mechanism and intervention research.

The results of the OFT revealed a high-stress state and anxiolytic-like behavior in the 5- and 14-day groups, while the EPM confirmed evident anxiety in the 21-day group. The OFT is a classic test to evaluate the locomotion and exploration behavior of rodents[38].The time spent in the central area reflects exploration and risky behavior. Rats prefer to walk close to the walls in an open field environment, a behavior called thigmotaxis[39]. Non-emotional rats prefer to leave the central area quickly, increased time spent in the central area reflected anxiety in rodents [40]. In our study, the time spent in the central area increased in the 5- and 14-day groups, indicating anxiolytic-like behavior. Although no significant change was seen in the 21-day group compared with the control group, the EPM confirmed evident anxiety in 21-day group unlike 5- and 14- day groups. Given that the two tests are based on different mechanisms to evaluate anxiety, it may be related to the reason why the 21 days group rats did not show anxiety behavior during the exploration of the novel environment, and the anxiety in the EPM occurred in the long term MMPM. Thus, further research is still needed for deeper explanation of this result. The maximum continuous distance, total distance travelled and mean velocity reflect the locomotor activity. No significant difference was observed in the total distance travelled or mean velocity among the groups. However, the maximum continuous distance travelled by the 5-day group increased compared with the control group. The reason for this may be that the 5-day MMPM induced a high-stress and restless state, then induced the increased locomotor activity. Finally, no change in locomotor activity was observed in the 14- and 21-day groups, indicating less excitability and aggressiveness, and exhibited as diminished exploration. Together with the result of increasing central time in these two groups, the findings reveal that the rats in the 5- and 14-day group mainly exhibited a high-stress and anxiolytic-like behavior. Additionally, the emotion state was intended to be stable in the 21-day group, an inhibition state with less exploration rather than high-stress or over-stimulation.

The EPM test, a further evaluation of anxiety, was performed, and the results revealed evident anxiety in the 21-day group. The time spent and the frequency of entries in the open arms both decreased in the 21-day group, indicated evident anxiety produced by the 21 days of MMPM. However, no evidence showed significant anxiety in the 5- or 14-day group, which is not consist with the result in OFT. We consider this changes in 5- and 14-day groups during OFT is more like a consequence of over-stimulation and stress response after MMPM. Recover from this change may occur quickly. However, when the MMPM duration extended to 21 days, the stress response disappeared and the negative effect of central fatigue became substantive, and rats are hard to recover, the following results are consistent with this conclusion as well.

The 5- and 14-day MMPM groups largely induced a high-stress state, a finding that is supported by the high corticosterone level in the blood serum of these rats. Corticosterone is an early change after the CNS is stimulated, and this indicator changes very fast[41]. Increasing level of corticosterone reflects stress response, high-stress state of the rodents, which may be a consequence of the activity of the hypothalamic–pituitary–adrenal (HPA) axis[42]. In addition, a previous study suggested that the MMPM increases corticosterone level because it was a stressor to rodents[43], a factor that affects the modelling by the MMPM as well. In this study, the corticosterone level increased in both the 5- and 14-day groups compared with the control group, indicates a high-stress state and an altered HPA activity due to sleep deprivation and MMPM stimulation. Remarkably, no change in corticosterone level was observed in the 21-day group compared with the control group. We consider this recovery to be a result of long-term adaptation to MMPM, such that, in this study, we found that the stress response induced by MMPM is bidirectional. The 21-day MMPM modelling could significantly decrease or even remove the effect of the model itself as a stressor to rats, which could make it more reliable in constructing a central fatigue model.

The NMDA receptors are reportedly involved in synaptic plasticity and various psychiatric activities[44]. The hippocampus is the main brain region associated with cognition and synaptic plasticity during central fatigue[45]. NR1 and NR2A are two subunits of the N-methyl-d-aspartate receptor that regulate brain function in the hippocampus, including synapses signal transduction. This process have been found to be closely related to anxiety[26], central fatigue[15], and cognition[46]. Consistent with a previous study [38], our data showed that all the MMPM protocols down regulated the gene expression of the NR1 and NR2A in the hippocampus. However, only 21-day MMPM decreased the protein expression of these receptors, this result mainly concerns the signal pathway affected by the NR1/NR2A gene change, the 5- or 14-day MMPM did not affect the protein formation until 21 days.

If the down-regulation of NMDAR is the initial change, the morphological changes may follow. We consider that the degenerative ultrastructural changes in the 21-day MMPM group are a consequence of long-term dysfunction of the synaptic plasticity, and the effects of central fatigue. As expected, the ultrastructural changes in the 5- and 14-day MMPM groups were inconspicuous. The mitochondria is known to be closely related to energy consumption and neuron function, it can be influenced and phenocopied by environmental factors[47]. Moreover, brain energy metabolism is closely related to central fatigue[48–49]. The 21-day MMPM degenerated the mitochondrial structure and morphology, whereas the 5- and 14-day durations did not show any evident changes. We consider that the effects of the 5- and 14-day MMPM were more of a psychophysiological condition, from which the animal can recover, and the 21-day MMPM could damage the energy mitochondrial metabolism system and function.

## 5. Conclusion

The MMPM can result in NR1 and NR2A gene down-regulation. However, different levels of brain dysfunction on rats depend on the duration of the modelling. The effect of 5 and 14 days of MMPM remains to be on stress response, indicating a psychophysiological condition from which the rats can recover. When the modelling lasted for 21 days, the rats exhibited anxiety and substantial degenerative changes in the hippocampal synapses and mitochondria. These changes may be related to the decrease of the NR1/NR2A proteins. Overall, subjection to the 21 days of MMPM can be an effective model of central fatigue.

## Supporting information

S1 File Raw data of OFT. (ZIP)

S2 File Raw data of EPM. (ZIP)

S3 File Raw data of corticosterone. (ZIP)

S4 File Raw data of NR1 and NR2A. (ZIP)

S5 File Graphpad prism data. (ZIP)

S6 File TEM pictures. (ZIP)
