# Supplementary figures and images for: Distinct behavioral and brain changes after different durations of the modified multiple platform method on rats: An animal model of central fatigue
Source: PLoS One. 2017 May 11;12(5):e0176850. doi: 10.1371/journal.pone.0176850 (PMC5426622; doi:10.1371/journal.pone.0176850)

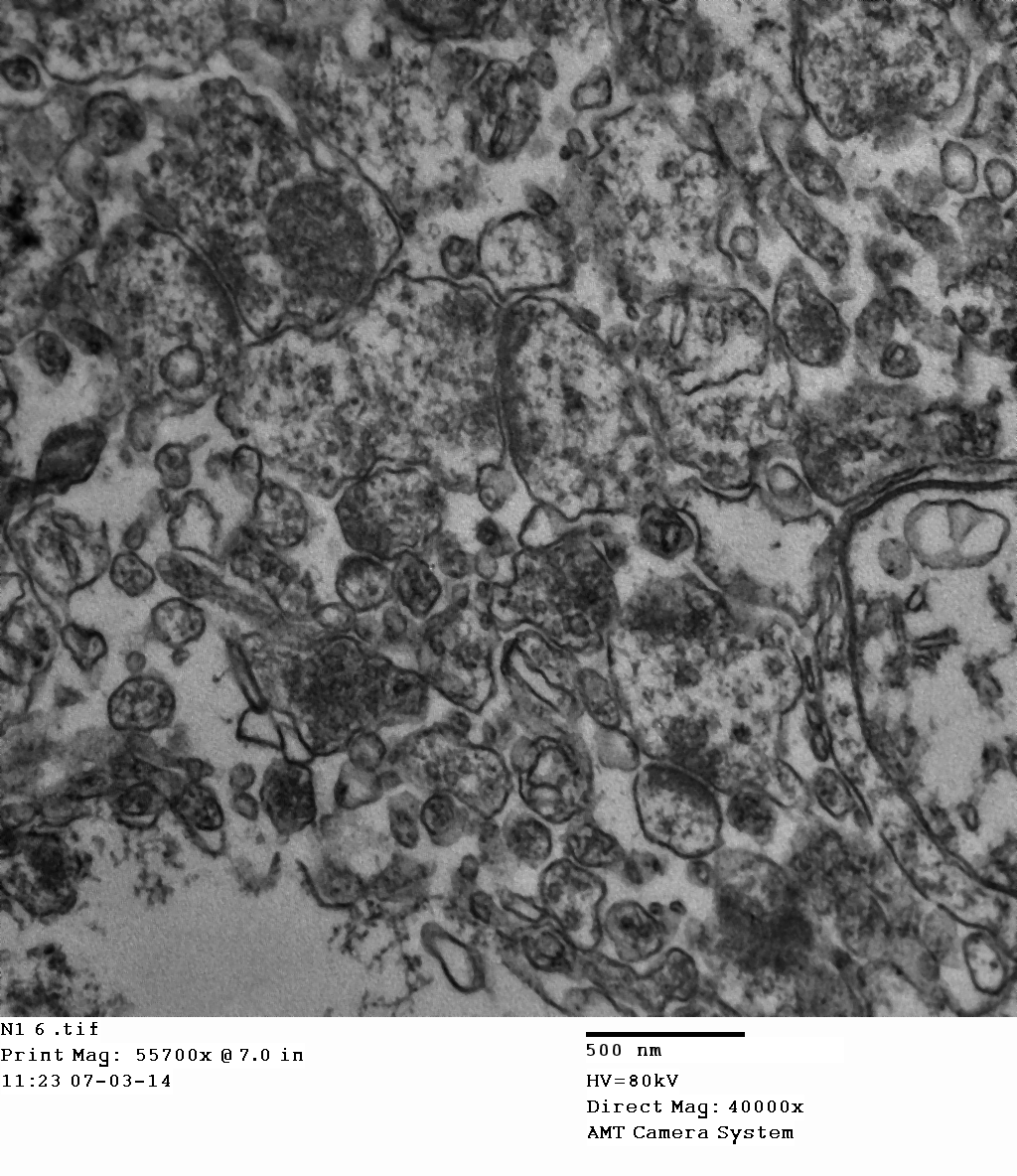

Supplement: S6 File — (ZIP) [file pone.0176850.s006.zip › S6 Original TEM pictures/figure6-A.tif]

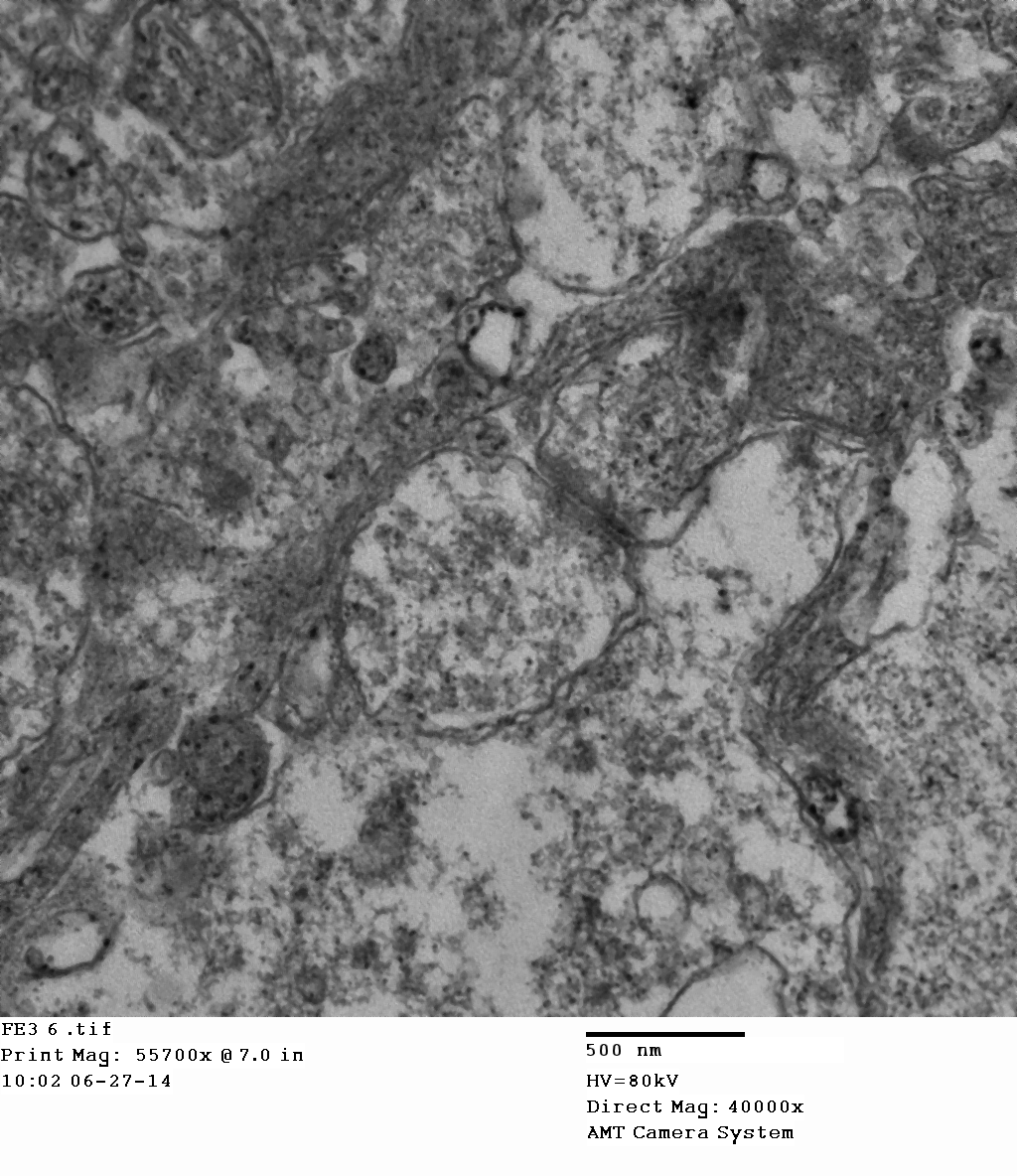

Supplement: S6 File — (ZIP) [file pone.0176850.s006.zip › S6 Original TEM pictures/figure6-B.tif]

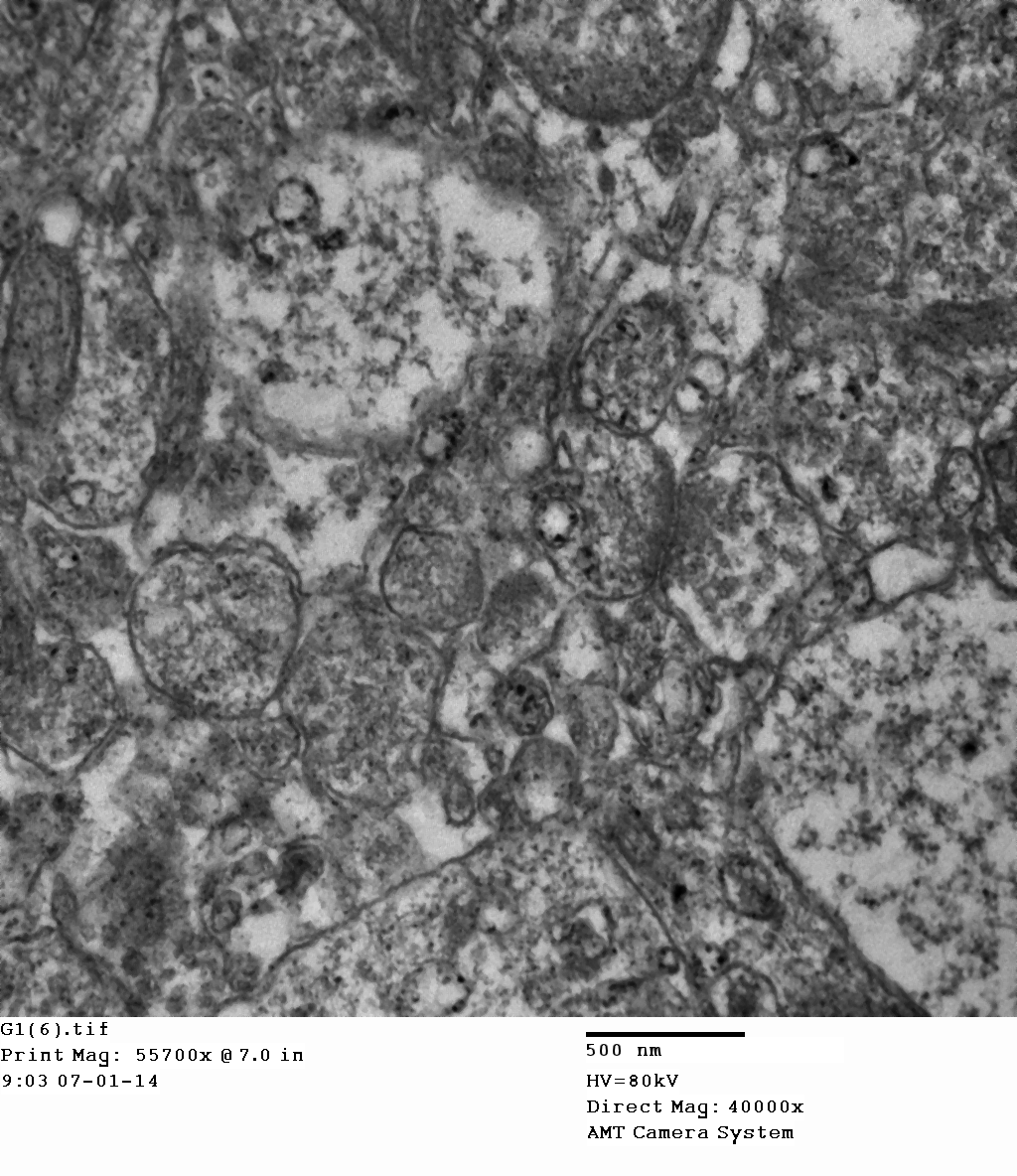

Supplement: S6 File — (ZIP) [file pone.0176850.s006.zip › S6 Original TEM pictures/figure6-C.tif]

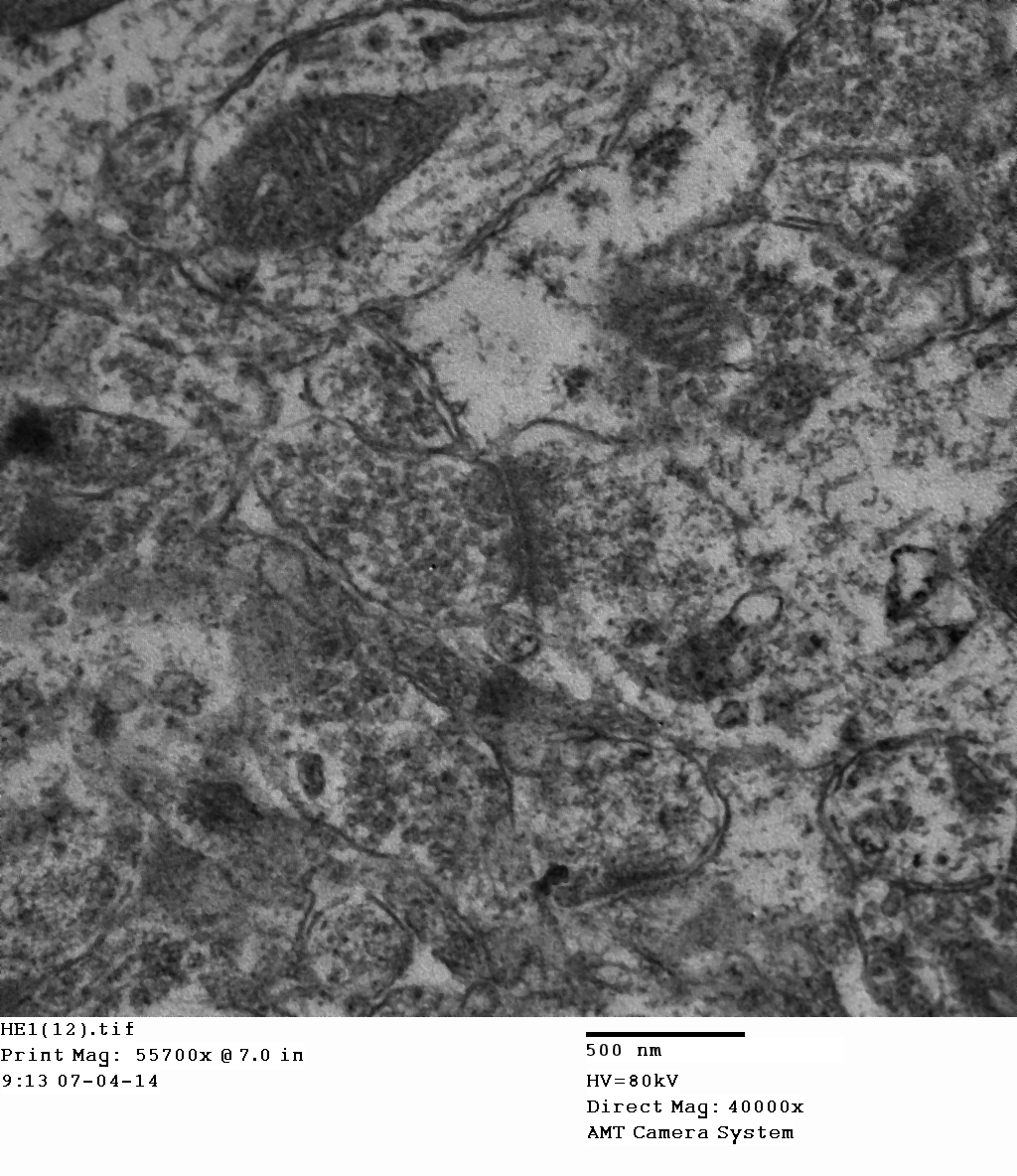

Supplement: S6 File — (ZIP) [file pone.0176850.s006.zip › S6 Original TEM pictures/figure6-D.tif]

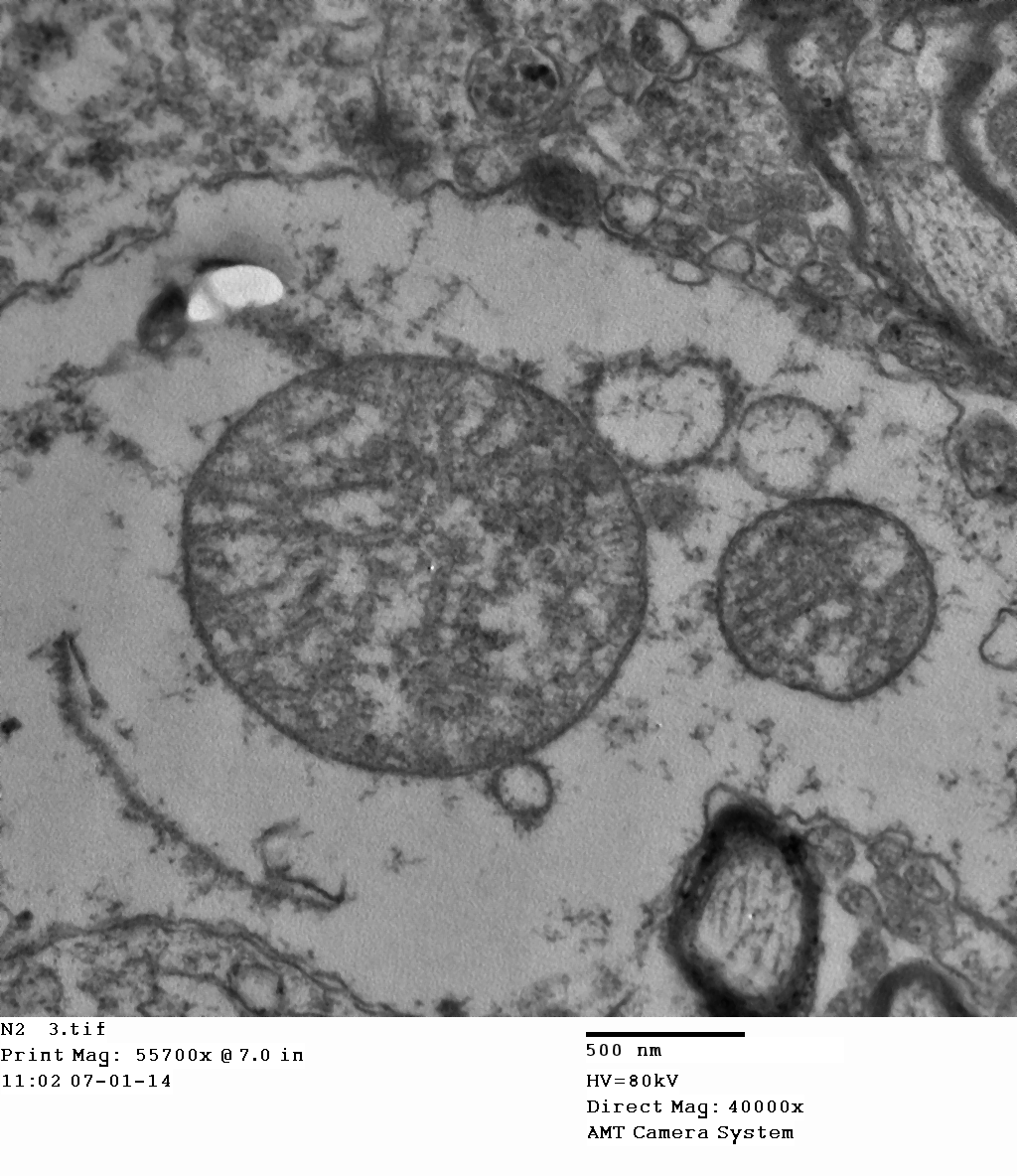

Supplement: S6 File — (ZIP) [file pone.0176850.s006.zip › S6 Original TEM pictures/figure7-A.tif]

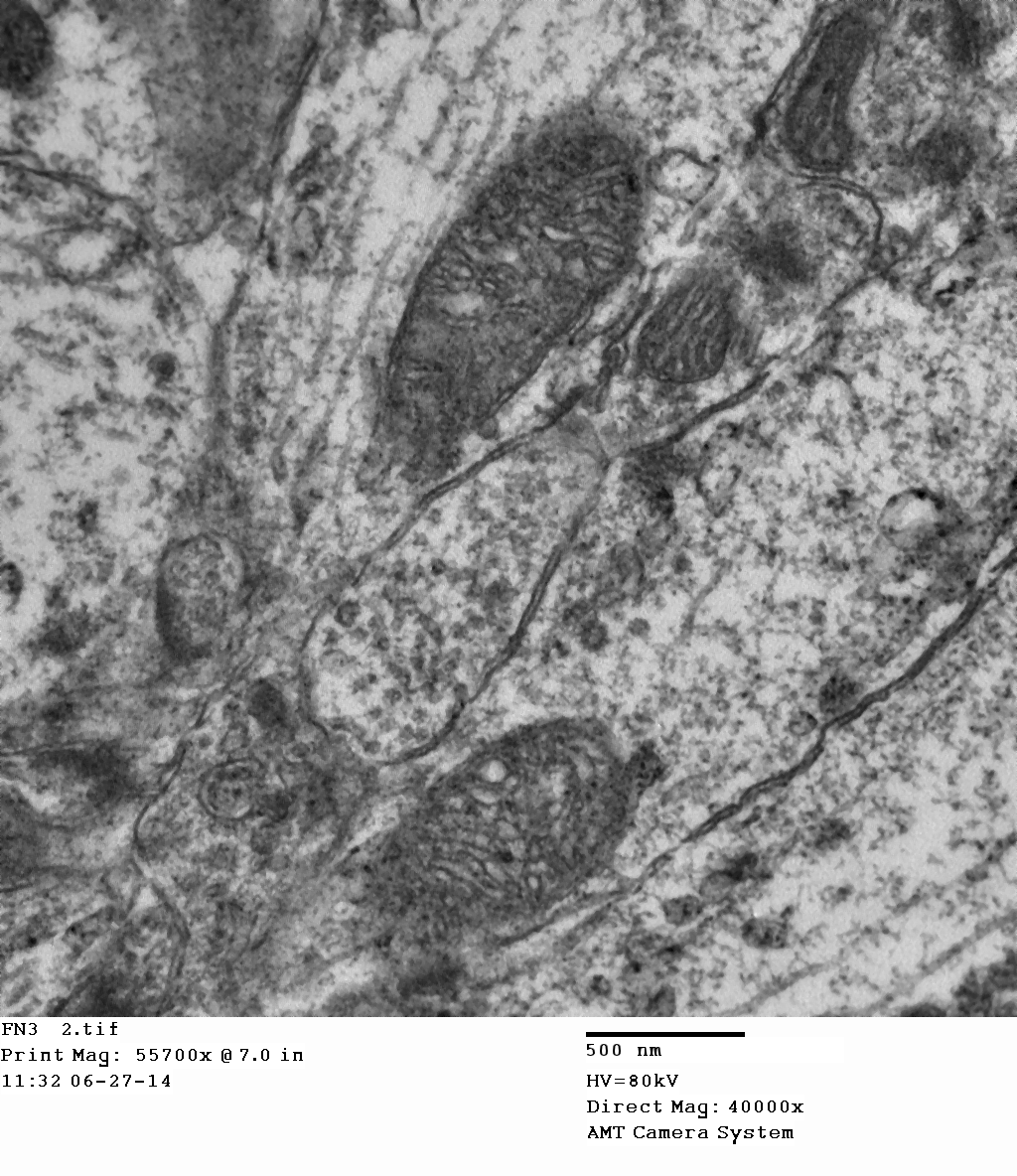

Supplement: S6 File — (ZIP) [file pone.0176850.s006.zip › S6 Original TEM pictures/figure7-B.tif]

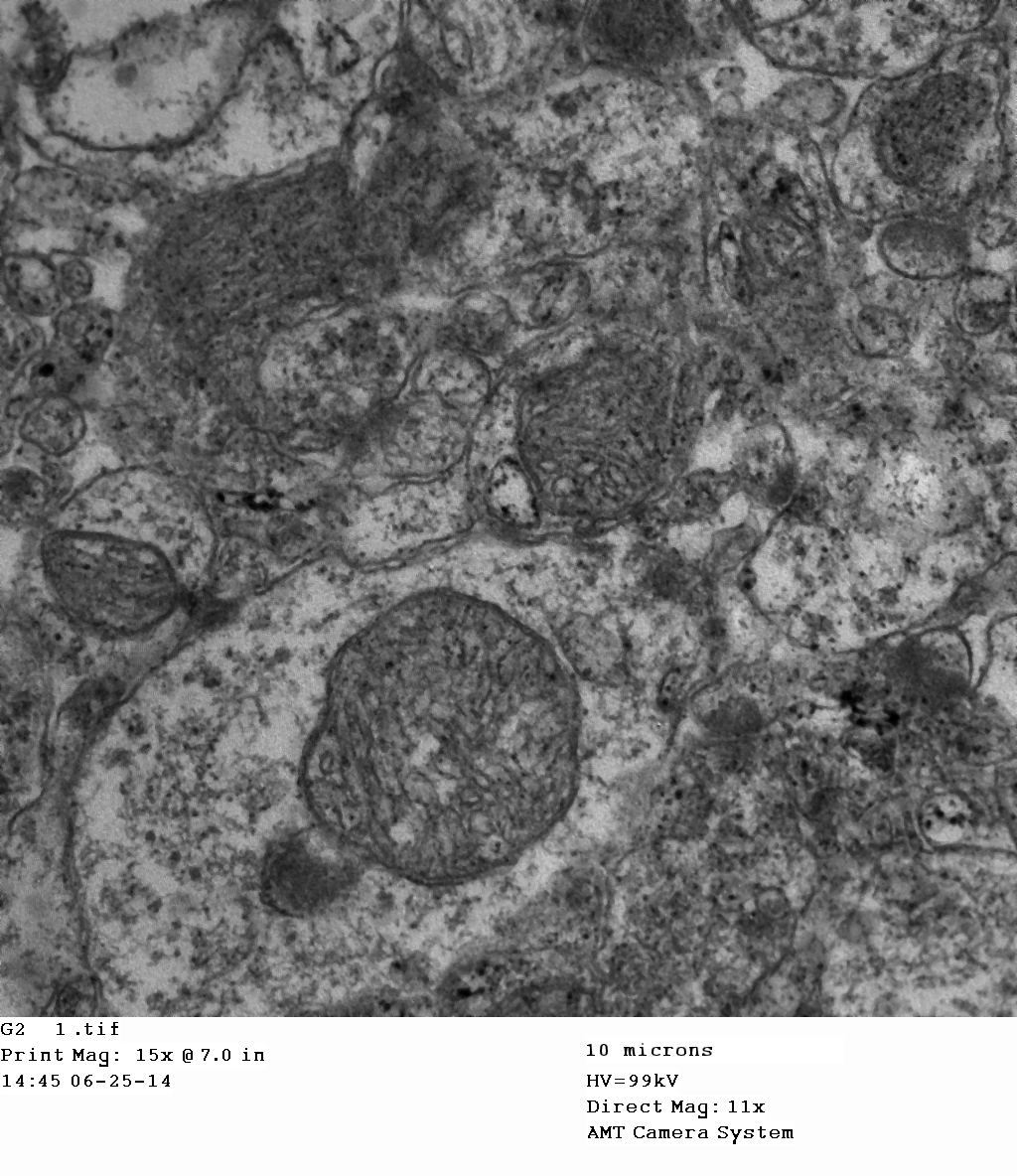

Supplement: S6 File — (ZIP) [file pone.0176850.s006.zip › S6 Original TEM pictures/figure7-C.tif]

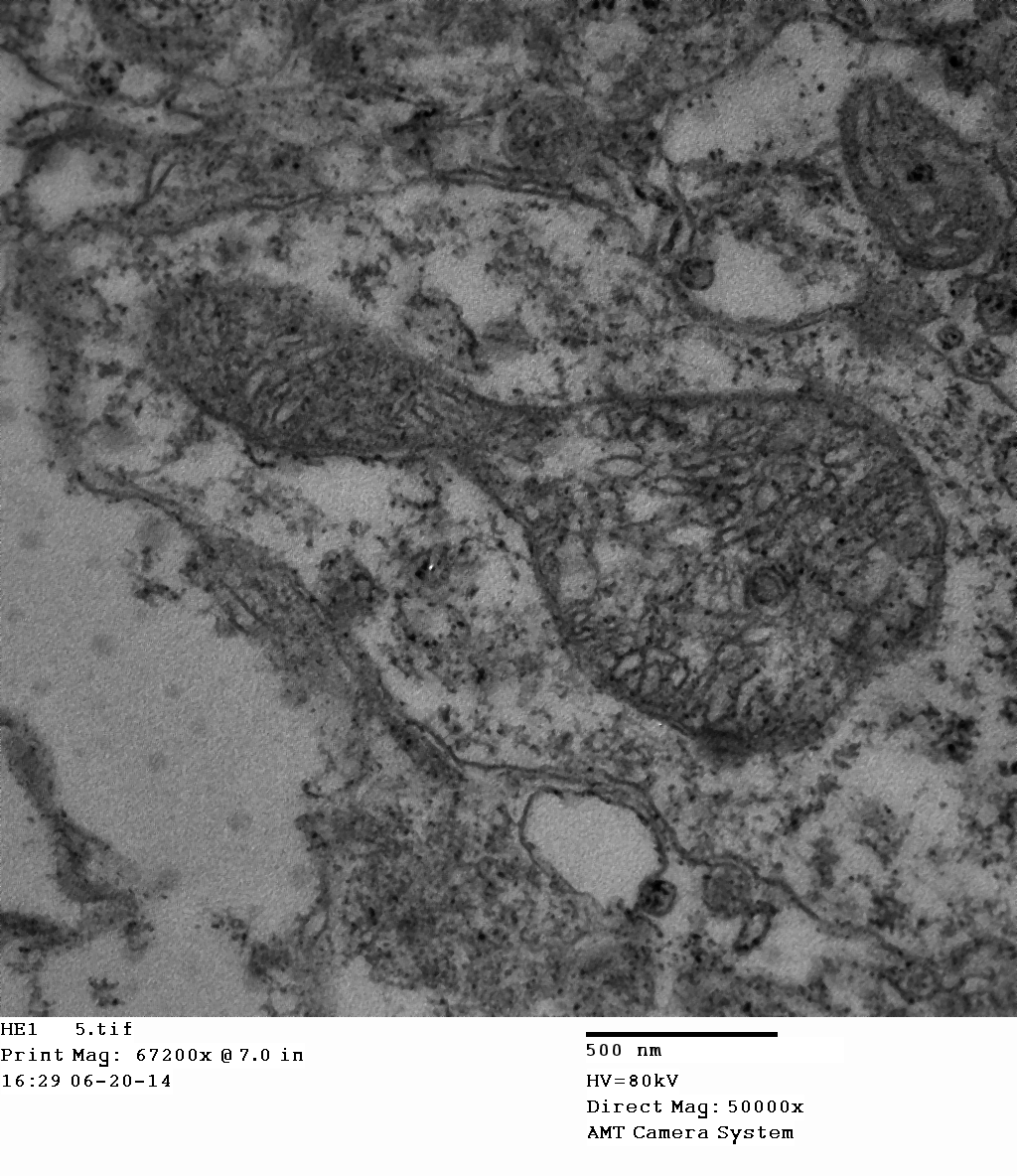

Supplement: S6 File — (ZIP) [file pone.0176850.s006.zip › S6 Original TEM pictures/figure7-D.tif]
